# Behavior and biomechanics: flapping frequency during tandem and solo flights of cliff swallows

**DOI:** 10.1242/jeb.249393

**Published:** 2025-01-02

**Authors:** Sophia Chizhikova, Laura X. Mendez, Tyson L. Hedrick

**Affiliations:** Department of Biology, University of North Carolina at Chapel Hill, Chapel Hill, NC 27510, USA

**Keywords:** Locomotion, Biomechanics, Wingbeat frequency, Kinematics, Birds

## Abstract

Aerodynamic models of bird flight, assuming power minimization, predict a quadratic relationship (i.e. U-shaped curve) between flapping frequency and airspeed. This relationship is supported by experimental bird flight data from wind tunnels, but the degree to which it characterizes natural flight, and the extent to which birds might modify it to accommodate other behaviors, is less known. We hypothesized that the U-shaped relationship would vary or vanish when minimizing power is not a primary consideration. We analyzed videos of wild cliff swallows (*Petrochelidon pyrrhonota*) engaged in solo and tandem (i.e. following or being followed by a conspecific) flights to collect bird flapping frequencies and airspeeds. Solo birds had a U-shaped flapping frequency to speed relationship. Birds engaged in tandem flights had the opposite pattern; their flapping frequencies varied with speed as an inverse U-shaped curve and were up to 2.1 times higher than solo birds at the same speed.

## Teaser

**Highlighted Article:** Compared with solo flight, cliff swallows flying in tandem with another bird have uniformly high flapping frequencies that are highest at intermediate speeds, the opposite of the solo pattern.

## INTRODUCTION

Flying animals are predicted to have a quadratic relationship between the muscle power required for flight and flight speed, arising from the underlying variation in aerodynamic power required to produce sufficient lift and overcome drag at different flight speeds (Rayner, 1999). Assuming that the mechanical work performed per flap is constant, variation in flapping frequency allows muscle power output to meet these aerodynamic requirements (Pennycuick, 2008). Together, these effects produce the expectation of a U-shaped relationship between flapping frequency and flight speed. Although historically contentious (Dial et al., 1997; Rayner, 1999), this expectation is now well-supported by laboratory wind tunnel studies where speed is under experimental control for solo flights (Ward et al., 2001; Tobalske et al., 2003; Guigueno et al., 2019) and by free-flight studies measuring flapping frequency and speed with animal-borne sensors (Usherwood et al., 2011). This variation in frequency even occurs when birds are probably not strictly meeting the constant work per wingbeat assumption, e.g. by co-varying flapping amplitude and frequency (Tobalske, 2000). Furthermore, changes in overall flapping frequency might be the result of one of two different processes: (1) varying the duration of each individual flap by changing duration of the upstroke portion of the flapping cycle (Hedrick et al., 2003) or (2) interspersing brief non-flapping periods among bouts of flapping (Bruderer et al., 2001). From the standpoint of this paper, both types of alteration have a similar effect on the average power output and can be considered equivalent. Despite this prior work, the extent to which the U-shaped flapping frequency to speed relationship is subject to modification to accommodate other behaviors during natural flight is less well understood. For example, data from different bird species suggest that flying in a group alters the flapping frequency to speed relationship (Usherwood et al., 2011; Corcoran and Hedrick, 2019; Ling et al., 2019; Taylor et al., 2019), possibly due to either aerodynamic interactions between birds or the need for birds to pay attention to and react to the movements of neighbors. Changes to flapping frequency among these different contexts ranged from approximately 1% to 18%. Because the U-shaped flapping frequency to flight speed relationship arises from the assumption that birds fly economically (i.e. minimize the energy cost per unit distance), we hypothesized that this relationship might substantially change or vanish in situations where minimizing the energetic cost of flight might not be the primary consideration, such as during intraspecific competition, foraging or predator escape.

To address this hypothesis, we used flapping frequency and flight kinematic data from field recordings of cliff swallows (*Petrochelidon pyrrhonota*) collected using high-speed 3D videography. Cliff swallows are gregarious birds that live in colonies with nests in proximity to one another. They engage in intraspecific nest parasitism, which occasionally results in flight behaviors, involving two birds, that greatly differ from regular flights (Brown and Brown, 1989). In this ‘tandem’ behavior, both birds participate in a chase-like flight, where one bird follows the other and copies its trajectory, which often includes complex evasion maneuvers. Following distances are small and the bouts even lead to occasional in-air physical contact. An earlier study of this behavior found that birds in tandem flight have identical flapping frequencies, hypothesized to allow for flapping phase matching between birds to enable a lower reaction latency to better evade or follow the other bird in a tandem pair (Shelton et al., 2014). Because tandem flight birds appear to alter their flapping frequency for reasons unrelated to the energetic cost of flight, we predicted that cliff swallows would exhibit a more U-shaped relationship in solo flights than during tandem flights. Although cliff swallows have not been subject to wind tunnel studies, closely related barn swallows do exhibit a U-shaped flapping frequency to speed relationship in solo flights (Park et al., 2001).

## MATERIALS AND METHODS

### Data collection

**Fig. 1.**
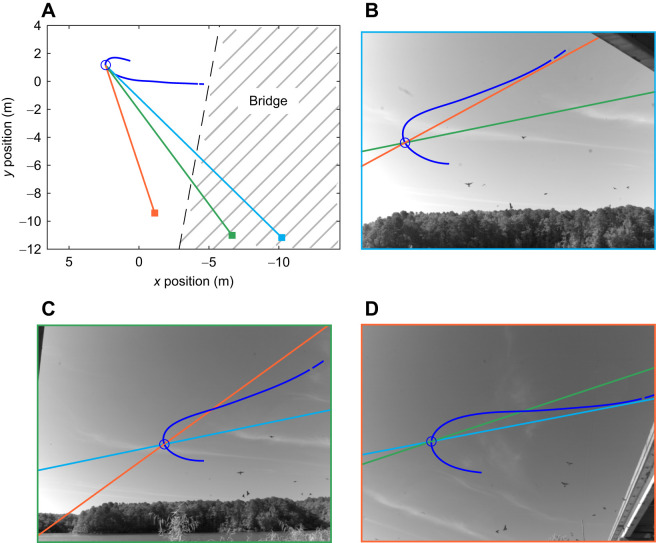
**Camera setup at Jordan Lake field site.** (A) Overhead view of the recording setup. Three cameras, represented by orange, green and blue squares, are shown at varying locations near the colony, along with each camera's line of sight extending towards a point in an example bird trajectory (dark blue line). (B–D) Views of the example bird's trajectory (dark blue) seen through each of the cameras (B, blue in A; C, green in A; D, orange in A), including the lines of sight of the other two cameras.

We analyzed videos of cliff swallow (*Petrochelidon pyrrhonota* Vieillot 1817) flights to collect wingbeat and kinematic data. The videos were recorded at a North Carolina Highway 751 bridge over Jordan Lake (35°49′42″N, 78°57′51″W). A cliff swallow colony of approximately 60 adults nested underneath the bridge from May to August during each recording year. The field videos were recorded from three cameras (NR5-S1, Integrated Design Tools, Pasadena, CA, USA) at a frequency of 100 Hz for a duration of up to 4.75 s (Fig. 1). All cameras were calibrated by passing an object of known length through the shared viewing volume, enabling measurement of the birds' 3D trajectories (Theriault et al., 2014). Videos were collected on 13 different days in 2012 and 2013 (Table S1) during low-wind conditions as revealed by the unrippled lake surface. Most tandem flight data were part of a previous analyses of maneuvering dynamics and tracking strategy (Shelton et al., 2014) and leader–follower relationships (Mwaffo et al., 2018). These data were re-examined for this study to ensure compatibility with newly analyzed recordings of solo flight behavior.

### Kinematic data processing and analysis

We used DLTdv, a video annotation tool, to analyze and compile 3D positions of individual birds recorded (Hedrick, 2008). To obtain 3D positions of tandem birds, we reviewed previous manual tracking coordinates from Shelton et al. (2014). For solo bird 3D positions, we used a custom automated tracker derived from prior work (Evangelista et al., 2017), followed by manual review. In all cases the tracking output produced a time series of 3D position data. These tracks were smoothed with a 6 Hz low-pass zero-lag digital filter to remove the effects of digitizing error and body oscillations due to flapping. Because flapping frequency is expected to vary with flight speed and other kinematic quantities such as acceleration and potential energy gain or loss, we calculated the instantaneous speed, acceleration, kinetic and potential energy, rate of change of kinetic and potential energy, and centripetal force for each tracked flight. Flight speed, *s*, was calculated as:
(1)

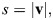
where **v** is the velocity vector 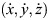. The instantaneous radius of curvature of the flight path, *R*, was calculated as:
(2)

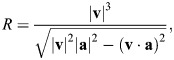
where **a** is the acceleration vector 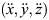. Centripetal acceleration, *F*, was then calculated as:
(3)

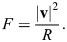
The mass-specific rate of change in potential energy, termed potential power here, was:
(4)

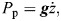
where ***g*** is the magnitude of gravitational acceleration. Similarly, the mass-specific rate of change in kinetic energy (termed kinetic power here) was:
(5)

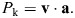


Note that Eqns 4–5 produce mass-specific power outputs (W kg^−1^). Frames with an absolute value of *P*_p_ or *P*_k_ greater than the 95th percentile for those data were dropped prior to further analysis as outliers. Flight speeds approaching zero violate assumptions made in the aerodynamic models that produce the U-shaped prediction, so frames with flight speed less than 2.5 m s^−1^ were also removed from the dataset.

### Wingbeat frequency analysis

To record wingbeat frequency we manually recorded the frame number of the full wing extension following upstroke for each flapping cycle. The amount of time between these frames was used to obtain the instantaneous wingbeat frequency *f* for each individual bird. Periods of gliding between flaps were included in the calculations, e.g. a 3 s period of gliding between two flaps has an estimated frequency of 1/3 Hz.

### Environmental data

Local environmental conditions were not recorded to the same standard for all video recording days, so we used meteorological records as a measure of environmental conditions, including temperature and wind speed (Table S1).

### Statistical data analysis

Bearing in mind that the swallow flights are dynamic, with repeated maneuvers and changes in flight speed and energy state (Fig. S1), track averages cannot represent the underlying non-linear flapping frequency to speed relationship. Thus, the underlying dataset for analysis was composed of samples (i.e. video frames) nested within flight tracks nested within recording days. We used linear mixed-effect models to account for the pseudoreplication within this dataset by including random effects for each separate flight track and each recording day. Linear mixed-effects fitting procedures provide a *t*-statistic for the fixed effects that accounts for the influence of the random effects but must make assumptions about the number of degrees of freedom to estimate significance (Baayen et al., 2008). This degrees of freedom estimate is often an upper bound calculated as the total number of observations minus the number of fixed effects. Because here we have many samples per individual bird track relative to the number of tracks recorded, we calculated *P*-values and 95% confidence intervals using the more conservative degrees of freedom estimate of number of tracks minus the number of fixed effects. The fixed effects included in our analysis were categorical flight behavior type (tandem or solo) along with flight speed, speed^2^, kinetic power, potential power, centripetal acceleration and environmental conditions. Random effects included a unique track identifier and the recording date. We first looked at both flight behaviors together (tandem and solo), and then at each behavior independently.

## RESULTS AND DISCUSSION

The methods described above were successfully used to record and process 119 solo and 30 tandem flight tracks (from 15 tandem flights) on 13 different recording days (Table S1). Example tandem and solo data are shown in Fig. S1.

### Distribution of observed flight speeds

We examined the flight speed distribution (Fig. S2) to see whether it was affected by participation in tandem behavior. Solo birds were sampled at flight speeds ranging between 2.5 and 15 m s^−1^, after applying the minimum speed threshold described earlier. Solo birds had an overall mean flight speed of 6.61 m s^−1^, while tandem birds had a mean of 6.81 m s^−1^. These means were not significantly different (*P*=0.31) according to a linear mixed-effects model of flight speed with tandem flight as a fixed effect and random effects of track identifier and recording day. The modal flight speed (with 1 m s^−1^ granularity) was 6 m s^−1^ for both flight behaviors (Fig. S2). Thus, while the competitive appearance of tandem flights might produce the expectation that they would exhibit faster flight speeds, that expectation is not supported by our data.

### Wingbeat frequency

**Fig. 2.**
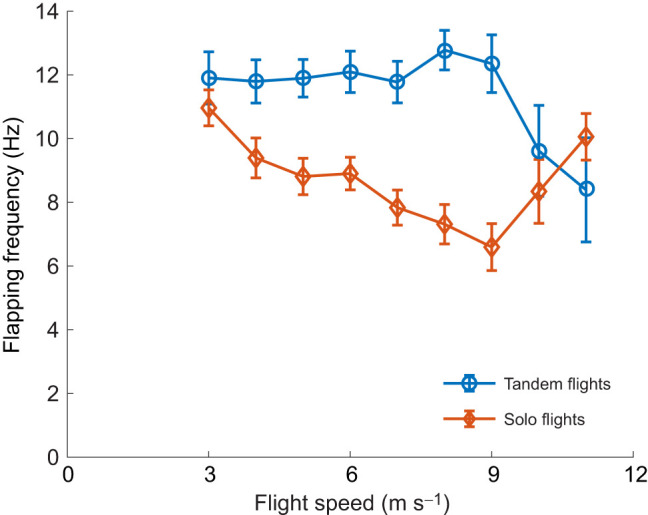
**Wingbeat frequency versus flight speed relationship of cliff swallows in tandem and solo flights.** The flapping frequencies from instantaneous samples were binned in 1 m s^−1^ increments by the corresponding instantaneous flight speed and then averaged to create these results. Error bars show standard error computed from the number of separate flight tracks contributing data to that speed bin. These results are not corrected for other factors such as kinetic or potential energy gain (or loss) that also affect wingbeat frequency. Only speed bins that include data from more than 5 tracks are shown. Sample sizes (i.e. track counts) were, from left to right, (10, 18, 22, 25, 27, 22, 16, 11, 7) for tandem flights and (32, 51, 65, 76, 74, 59, 36, 18, 9) for solo birds.

Without considering the effect of other kinematic parameters such as gain (or loss) of kinetic and potential energy, we found that the participation in tandem behavior substantially affected the relationship between wingbeat frequency and flight speed in cliff swallows (Fig. 2). Tandem flights tended to have higher frequencies than solo flights. For example, at a flight speed of 9 m s^−1^ tandem birds used a frequency 112% higher than solo birds (12.35 versus 5.83 Hz). Solo flight data exhibited the hypothesized upwardly concave U-shaped curve, while tandem flight data had a downwardly concave shape with the highest flapping frequencies at intermediate flight speeds.

To assess whether differences in other kinematic parameters such as change in energy state or centripetal acceleration could account for the differences between tandem and solo flights, we first examined all tandem and solo flight data in a linear mixed-effects model with tandem flight behavior as a categorical variable, along with other continuous kinematic measurements and environmental variables for each recording day (Table 1). In this analysis, the tandem categorical designation was significant (*P*<0.001; Table 1) and had a positive coefficient indicating that tandem flights used an approximately 2.5 Hz higher flapping frequency than solo flights after accounting for the other factors in the model. Thus, tandem and solo flights have significantly different relationships between flight speed and flapping frequency. Centripetal acceleration, kinetic power and potential power were all also significantly and positively associated with flapping frequency (Table 1). Flight speed was not significantly associated with flapping frequency over this dataset, while flight speed squared was marginally significant with an effect size less than that of tandem versus solo behavior. The environmental factors of average daily temperature and average daily wind speed were not significant (*P*=0.542 and *P*=0.243, respectively) when included in the linear mixed-effects model of all flights and were therefore not included in the final analysis.

**Table 1.** Results from linear mixed-effects statistical models for flapping frequency fit to three datasets

Statistical model term	All flights (*n*=149 tracks)		Solo flights (*n*=119)		Tandem flights (*n*=30)
Intercept	7.79 (6.57, 9.01)		10.69 (9.56, 11.81)		8.81 (7.21, 10.42)
	*P*<0.001	* *	*P*<0.001	* *	*P*<0.001
*t* (1 if tandem, 0 if solo)	2.59 (1.53, 3.66)		n.a.		n.a.
* *	*P*<0.001	* *			
*s* (speed, m s^−1^)	−0.060 (−0.197, 0.078)		−0.936 (−1.149, −0.722)		0.700 (0.533, 0.868)
* *	*P*=0.390	* *	*P*<0.001	* *	*P*<0.001
*s*^2^ (speed^2^, m^2^ s^−2^)	0.010 (0.0004, 0.020)		0.073 (0.057, 0.088)		−0.043 (−0.055, −0.031)
* *	*P*=0.042	* *	*P*<0.001	* *	*P*<0.001
*F* (centripetal acceleration, m s^−2^)	0.022 (0.018, 0.027)		0.024 (0.017, 0.030)		0.018 (0.013, 0.024)
* *	*P*<0.001	* *	*P*<0.001	* *	*P*<0.001
*P*_k_ (kinetic power, W kg^−1^)	0.003 (0.003, 0.004)		0.004 (0.003, 0.006)		0.002 (0.001, 0.003)
* *	*P*<0.001	* *	*P*<0.001	* *	*P*<0.001
*P*_p_ (potential power, W kg^−1^)	0.043 (0.039, 0.046)		0.069 (0.063, 0.075)		0.022 (0.018–0.026)
* *	*P*<0.001	* *	*P*<0.001	* *	*P*<0.001

Values are mean with 95% confidence intervals (in parentheses). Calculation of *P*-values and confidence intervals use the number of tracks to determine the degrees of freedom for the model. n.a., not available.

LME formula: frequency ∼1+t+s+s^2^+F+P_p_+P_k_+ (1|trackID)+(1|recordingDate).

We next analyzed the solo flight data separately, using the same mixed-effects model as described above (except for the tandem term). Detailed results are provided in Table 1; all fixed effects were highly significant (*P*<0.001). Flight speed had a positive coefficient while flight speed squared had a negative coefficient, a pairing that produces an upwardly concave or U-shaped curve.

Lastly, we analyzed the tandem flight data separately, again using the mixed-effects model described above. All terms were highly significant (*P*<0.001, Table 1) and coefficient magnitudes for changes in kinetic and potential energy as well as centripetal acceleration were consistent with earlier results. The coefficients related to flight speed and flight speed squared were similar in magnitude but opposite in sign compared with those from solo flights, confirming a downwardly concave relationship between flapping frequency and flight speed for birds in tandem flights.

### Conclusions and implications

As described above, our field measurements of instantaneous flapping frequency and flight speed in cliff swallows revealed that during solo flight, these birds exhibited the hypothesized U-shaped flapping frequency to speed relationship. However, when following or being followed by a conspecific (i.e. tandem flight), flapping frequency was on average higher than in solo flight and the flapping frequency to speed relationship had an inverse U-shape, with the highest frequencies occurring at intermediate flight speeds. The observed differences in flapping frequency between solo and tandem flights are also much greater in magnitude than previously described behavioral effects of flapping frequency. Here, we found tandem flight flapping frequencies were up to 112% greater than solo flight flapping frequencies at the same speed, while the superficially similar context of homing pigeons flying in pairs versus solo (Taylor et al., 2019) exhibited only an 18% increase associated with group behavior. Flocking flight contexts in pigeons were associated with a flapping frequency increase of only 0.1 Hz, or about 1.5% (Usherwood et al., 2011), whereas Corcoran and Hedrick (2019) report similarly small effects of flock position and neighbor distance on flapping frequency in a variety of shorebird species. Thus, while all these studies demonstrate that birds can and do alter their flight speed to flapping frequency relationship, the highly varied outcomes for different contexts suggest that the factors driving these differences may also be quite distinct.

The flapping frequency difference between tandem and solo flight behavior persisted even after accounting for the effect of maneuvers and changes in kinetic or potential energy on flapping frequency and thus must have a different explanation. Shelton et al. (2014) found that the trailing bird in a tandem pair avoided flying in the wake of the lead bird, and that the leader and follower used the same flapping frequency. Therefore, aerodynamic interactions are not a likely explanation for the difference in flapping frequency between tandem and solo flights. Instead, we suggest that these differences result from differences in the importance of flight economy among the solo and tandem contexts. Shelton et al. (2014) also found that swallows in tandem flight phase-matched their flapping cycles and hypothesized that this allowed the birds to minimize biomechanical lag when following the maneuvers of their companion. We suggest a similar explanation for the high overall flapping frequency of tandem flights compared with solo flights: if each wingbeat provides a new opportunity for a muscle powered flight maneuver, increasing the number of flaps per second increases agility (Usherwood et al., 2011; Taylor et al., 2019), which is probably a valuable trait during competitive aerial interactions (Dakin et al., 2018). Taylor et al. (2019) also suggested that higher flapping frequencies allow for better gaze stabilization and visual tracking of nearby conspecifics, which is also important during aerial interactions.

We initially expected that swallows in the tandem flight context would exhibit less (or no) variation in flapping frequency with flight speed compared to solo flyers. Instead, our data showed that tandem flight birds have a downwardly concave flapping frequency to speed relationship that is opposite that of solo flight birds; tandem birds exhibited the highest flapping frequency at the speeds near to where solo birds had their lowest frequency. We hypothesize that this occurred because elevating flapping frequency could decrease locomotor efficiency (W kg^−1^ m^−1^), and that the speeds with the lowest solo flight flapping frequencies were also the lowest-cost flight speeds where the birds had the most scope for operating inefficiently.

Although energetic considerations underpin the expectation of a U-shaped flapping frequency to flight speed relationship, the precise energetic penalty due to departures from that expectation as seen here in tandem flights is unknown. High frequency flapping could reduce aerodynamic efficiency (i.e. lift to drag ratio) by altering parameters such as the reduced frequency (Spedding, 1992) or Strouhal number (Nudds et al., 2004). However, flapping amplitude was not measured in this study and in principle, variation in flapping amplitude could make reduced frequency and Strouhal number similar between tandem and solo flights despite the differences in flapping frequency. Flapping at high frequency would also increase the inertial costs of accelerating the wing since these costs increase with the third power of frequency, assuming amplitude is kept constant. However, these costs may be recovered in part or whole either by transfer to aerodynamic power requirements (Hedrick et al., 2004) or by elastic storage and release (Ingersoll and Lentink, 2018). Furthermore, a high flapping frequency may impact muscle efficiency in converting chemical energy to mechanical work, with higher flapping frequencies incurring more muscle activation costs, but the magnitude of this effect is unknown. Bruderer et al. (2001) remark that, based on detailed flapping kinematics from a wind tunnel study, barn swallows appear to use a wide range of muscle shortening velocities, suggesting that constraints related to muscle properties might be less applicable to that species and the closely related cliff swallows studied here. Despite the caveats associated with aerodynamic, inertial and muscle-based reductions in flight efficiency, we suggest that they all might nevertheless become important at sufficiently high flapping frequencies.

In summary, we found that cliff swallows exhibit substantial behavioral plasticity in flapping frequency related to the flight behavior context, demonstrating that the relationships between flapping frequency and flight speed predicted from theory and supported by diverse experiments and free flight recordings are not immutable, and birds adopt disparate flapping frequencies as the need arises during natural behavior. In this case, we hypothesize that the departure occurs because the birds favor agility over economy when engaged in tandem flight interactions with conspecifics. We expect that additional departures would also occur in other cases where agility is favored over economy such as prey capture, predator evasion and mating displays.

## Supplementary Material

10.1242/jexbio.249393_sup1 Supplementary information
