# Supplementary material for: Behavior and biomechanics: flapping frequency during tandem and solo flights of cliff swallows
Source: J Exp Biol. 2025 Jan 2;228(1):jeb249393. doi: 10.1242/jeb.249393 (PMC11708819; doi:10.1242/jeb.249393)
Supplement: Supplementary information [file jexbio-228-249393-s1.pdf]

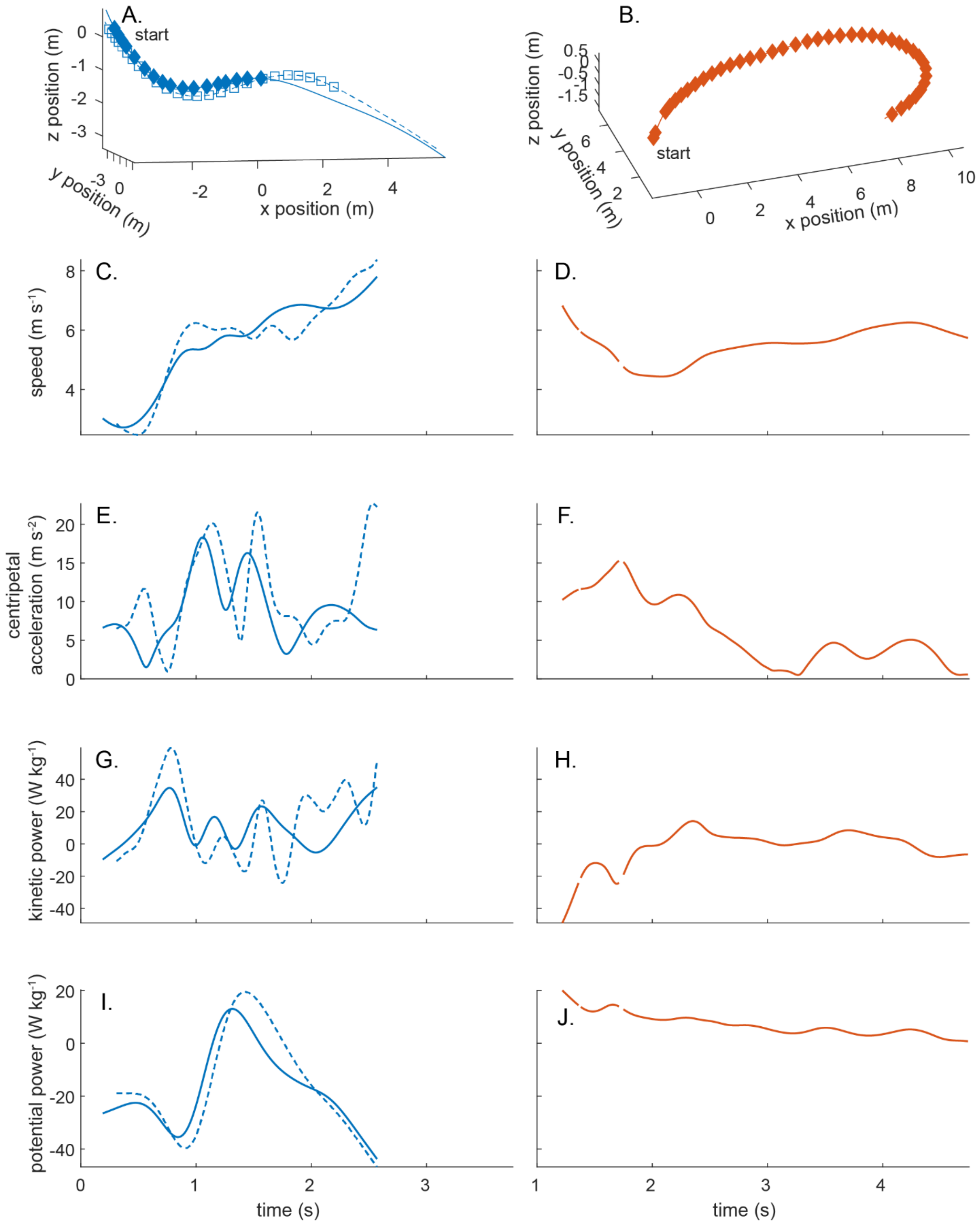

Fig. S1. Example tandem and solo flight data. The left column shows data from a tandem trial with the lead bird denoted as a solid line and the follower a dashed line. The right column from a solo trial. A,B. 3D trajectory was created for each bird by combining the x, y, and z position coordinates from three cameras. Markers show the start of a downstroke. C,D. flight speed s, E,F. centripetal acceleration F, G,H. kinetic power P_k, H,I. potential power P_p.

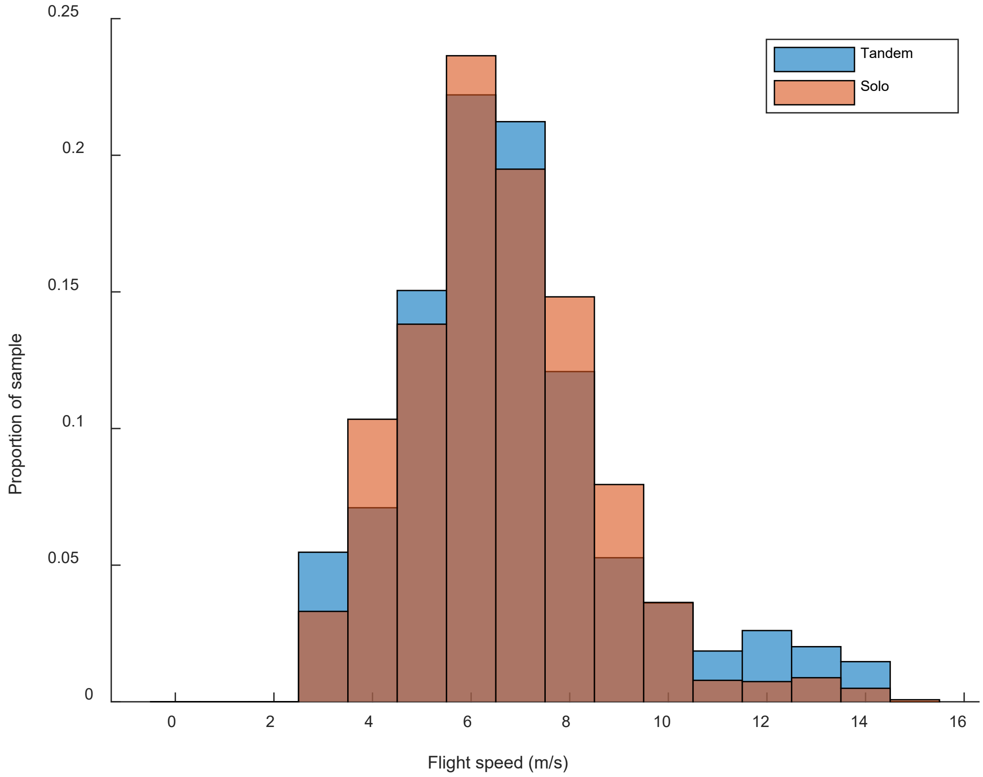

Fig. S2. Histogram of flight speed distribution for the solo (red) and tandem (blue) Cliff Swallow data sets. The speed distribution shows the proportion of the tandem or solo sample flying at a mean flight speed, plotted by 1 m/s intervals. Data points with flight speeds below 2.5 m/s were removed from the dataset because they do not meet theassumptions of the aerodynamic models used as a basis for predicting U-shaped flapping frequency to speed curves.

Table S1. Recording days, mean weather conditions by day, and number of flight trajectories collected.

Recording day (YYYY-MM-DD)	Total number of trajectories	Tandem trajectories	Solo trajectories	Mean wind speed (m s^{-1})	Mean temperature (°C)
**2012-05-07**	2	2	0	2.21	19.44
**2012-06-14**	19	2	17	3.03	20.72
**2012-06-15**	18	2	16	3.29	20.89
**2012-06-18**	19	2	17	2.62	22.11
**2012-06-19**	32	4	28	2.73	25.78
**2012-07-05**	4	0	4	2.01	28.22
**2012-07-06**	2	0	2	2.06	28.00
**2013-05-13**	8	0	8	1.54	13.56
**2013-05-14**	11	6	5	1.49	12.83
**2013-05-16**	15	4	11	3.14	23.56
**2013-05-17**	5	2	3	1.29	23.22
**2013-05-28**	7	2	5	3.71	22.50
**2013-05-29**	7	4	3	4.01	23.61
